# Klotho, Oxidative Stress, and Mitochondrial Damage in Kidney Disease

**DOI:** 10.3390/antiox12020239

**Published:** 2023-01-20

**Authors:** Javier Donate-Correa, Beatriz Martín-Carro, Jorge B. Cannata-Andía, Carmen Mora-Fernández, Juan F. Navarro-González

**Affiliations:** 1 Unidad de Investigación, Hospital Universitario Nuestra Señora de Candelaria, 38010 Santa Cruz de Tenerife, Spain; 2 GEENDIAB (Grupo Español para el Estudio de la Nefropatía Diabética), Sociedad Española de Nefrología, 39008 Santander, Spain; 3 Instituto de Tecnologías Biomédicas, Universidad de La Laguna, 38010 San Cristóbal de La Laguna, Spain; 4 RICORS2040 (RD21/0005/0013), Instituto de Salud Carlos III, 28029 Madrid, Spain; 5 RICORS2040 (RD21/0005/0019), Instituto de Salud Carlos III, 28029 Madrid, Spain; 6 Bone and Mineral Research Unit, Instituto de Investigación Sanitaria del Principado de Asturias (ISPA), 33011 Oviedo, Spain; 7 Servicio de Nefrología, Hospital Universitario Nuestra Señora de Candelaria, 38010 Santa Cruz de Tenerife, Spain

**Keywords:** Klotho, oxidative stress, mitochondrial dysfunction, cellular senescence, kidney disease

## Abstract

Reducing oxidative stress stands at the center of a prevention and control strategy for mitigating cellular senescence and aging. Kidney disease is characterized by a premature aging syndrome, and to find a modulator targeting against oxidative stress, mitochondrial dysfunction, and cellular senescence in kidney cells could be of great significance to prevent and control the progression of this disease. This review focuses on the pathogenic mechanisms related to the appearance of oxidative stress damage and mitochondrial dysfunction in kidney disease. In this scenario, the anti-aging Klotho protein plays a crucial role by modulating signaling pathways involving the manganese-containing superoxide dismutase (Mn-SOD) and the transcription factors FoxO and Nrf2, known antioxidant systems, and other known mitochondrial function regulators, such as mitochondrial uncoupling protein 1 (UCP1), B-cell lymphoma-2 (BCL-2), Wnt/β-catenin, peroxisome proliferator-activated receptor gamma coactivator 1-alpha (PGC-1 alpha), transcription factor EB, (TFEB), and peroxisome proliferator-activated receptor gamma (PPAR-gamma). Therefore, Klotho is postulated as a very promising new target for future therapeutic strategies against oxidative stress, mitochondria abnormalities, and cellular senescence in kidney disease patients.

## 1. Introduction

The prevalence of chronic kidney disease (CKD) is rapidly rising worldwide and will become the fifth leading cause of death by 2040 [1,2]. Despite recent clinical trials showing the therapeutic potential of inhibiting renin-angiotensin-aldosterone system (RAS) and sodium-glucose cotransporter-2 (SGLT2) in slowing the progression of CKD [3,4], there is still a high incidence of end-stage renal disease (ESRD). The reason probably lies in the multifactorial mechanisms, not clinically determined in detail, that are responsible for CKD progression and lead to the premature aging syndrome that characterizes this disease [5].

The kidneys are highly metabolic organs that regulate the composition of body fluids through the filtration and reabsorption of various components. Among these processes, the one that demands the most energy is the active reabsorption of sodium, glucose, and other ions from the filtrate [6]. To accomplish this important primary function, tubular cells consume large amounts of adenosine triphosphate (ATP), which is generated exclusively by aerobic metabolism. This explains the high mitochondrial density and the high oxygen consumption in kidneys, which accounts for up to 10% of total body consumption [7]. However, highly energetic organs such as kidneys are more vulnerable to damage caused by oxidative stress.

Oxidative stress is defined as the increased production of reactive oxygen species (ROS) with a concomitant decrease in the antioxidant defense system [8]. Both the accumulation of ROS and the redox imbalance leads to the dysregulation and/or oxidation of proteins, nucleic acids, carbohydrates, and lipids, contributing to cell apoptosis and necrosis [9]. Dysregulation induced by oxidative stress ultimately leads to tissue damage, and has been implicated in the pathophysiology of acute and chronic kidney lesions and associated with kidney disease progression [10]. Thus, oxidative stress and mitochondrial deregulation, with an abundant production of ROS, have been observed even in early stages of kidney disease [11].

The correct functioning of the tubular cells is vital to maintain kidney activity and their dysfunction has harmful consequences [6]. Kidney tubular cells are particularly susceptible to oxidative stress which induces the appearance of a senescent phenotype [12,13,14,15]. Cell senescence refers to physiological, structural, biochemical, and molecular changes that occur progressively during aging, culminating in the permanent cessation of cell division. In tubular cells, senescence has been involved in acute kidney injury (AKI), AKI-to-CKD transition, CKD progression, transplant rejection, and aging [12,16,17]. The senescence processes in cells trigger the appearance of the senescence-associated secretory phenotype (SASP) [18,19]. SASP is characterized by the secretion of high levels of pro-inflammatory cytokines, immune modulators, growth factors, proteases, and profibrotic factors [17,20,21,22,23,24]. Although senescence can be beneficial by removing inactive cells through phagocytosis, contributing to tissue remodeling and damage resolution, persistent senescence-mediated inflammation can lead to aberrant tissue remodeling and fibrosis, which contributes to kidney dysfunction and to renal-damage progression [12,17,20,21,22,23].

**Figure 1:**
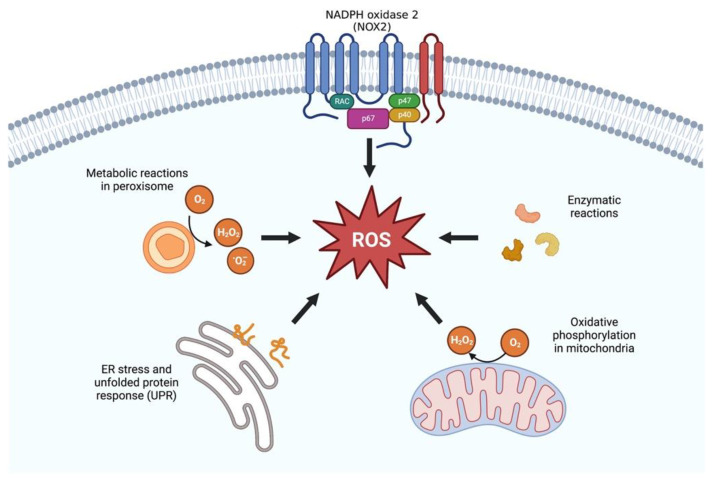
Endogenous reactive oxygen species (ROS) production. Intracellular ROS is primarily produced by the mitochondria, in which ROS are largely generated by the electron transport chain. Other sources of ROS include the transmembrane NADPH oxidases; xanthine oxidoreductase in peroxisomes; protein disulfide isomerase, which are involved in the unfolded protein response in the endoplasmic reticulum (ER); and diverse enzymatic reactions that, among others, include prostaglandin synthesis, auto-oxidation of adrenalin, and reduced riboflavin.

The mechanisms of endogenous ROS production include oxidative phosphorylation system in mitochondria—by far, the major source of ROS [24], endoplasmic reticulum stress and unfolded protein response, metabolic reactions in peroxisomes, NADPH oxidase 2 system, and enzymatic reactions (Figure 1). Recent studies have suggested that mitochondria abnormalities could be implicated in various kidney disorders including AKI, kidney fibrosis, and diabetic nephropathy [13,25,26,27,28,29,30], being intimately associated with the accumulation of senescent tubular cells, detected even in early stages of CKD [31] and with the progression of the disease [16,25,31]. Thus, these abnormalities not only result in decreased energy availability in tubular cells but also affect the whole kidney, leading to the production of oxidative stress [32] and, ultimately, to the appearance of SASP [16,23,32,33,34]. The leakage of electrons in the mitochondria constitutes the main source of ROS. Structurally, these organelles present an outer and inner membrane, the latter of which contains the oxidative phosphorylation system (OXPHOS). In this inner membrane, electrons derived from metabolic reducing equivalents (NADH and FADH2) undergo a passage throughout the electron transport chain electrons to reduce oxygen to water and produce ATP. During this process, a small percentage (0.4–4%) of electrons may “leak” from the respiratory chain (in particular at complexes I and III) and partially reduce oxygen, forming superoxide radicals [31]. The antioxidant manganese-containing superoxide dismutase (Mn-SOD) is expressed in the mitochondrial matrix and is a key enzyme in the detoxification of free radicals, keeping the redox balance stable [35].

Reducing oxidative stress and preserving mitochondrial health stands in the center of a prevention and control strategy for mitigating cellular senescence and aging [36,37]. Kidney disease is no exception and to find a modulator targeting against oxidative stress, mitochondrial dysfunction, and cellular senescence in kidney tubular cells could be of great significance to prevent and control the progression of kidney injury in CKD [38]. Thus, targeting mitochondria-derived oxidative stress could prevent and slow down the progression of CKD and minimize the development of severe systemic complications. Together with Mn-SOD, diverse oxidative stress-protective transcription factors, such as forkhead box class O (FoxO) and nuclear factor erythroid 2-related factor 2 (Nrf2), are promising targets to counteract ROS-mediated damage in several disorders including CKD [37,38,39].

**Figure 2:**
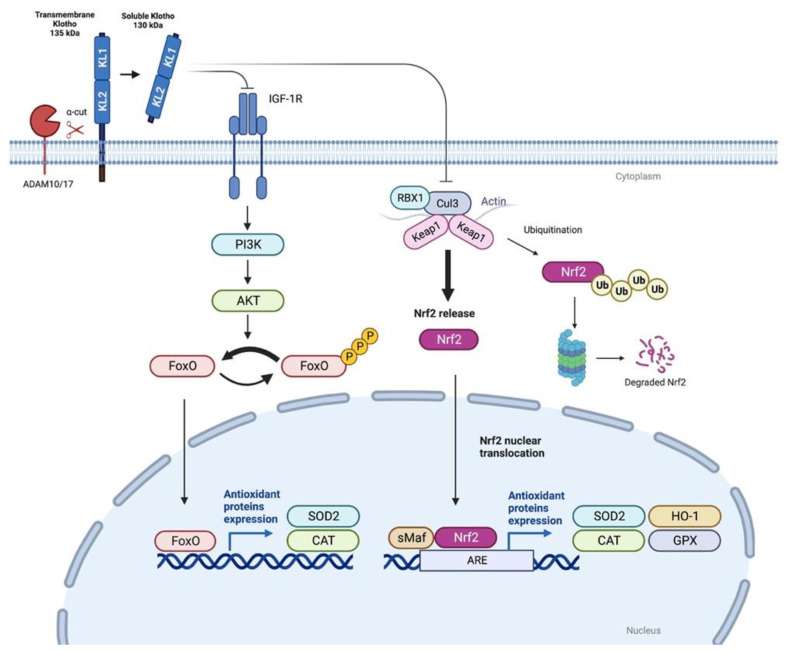
Soluble Klotho-mediated antioxidant mechanisms. Membrane Klotho can be cleaved by membrane-anchored secretases ADAM10/17, which release the extracellular domain into the extracellular space. Soluble Klotho decreases oxidative stress via the inhibition of insulin/IGF-1/PI3K/Akt/FoxO pathway, promoting the expression of superoxide dismutase (SOD2) and catalase (CAT). Similarly, soluble Klotho also reduces oxidative stress through the activation of the cellular protective Nrf2 pathway, which promotes the transcription of genes related to antioxidant defense, such as hemeoxygenase-1 (HO-1), SOD2, CAT, and glutathione peroxidase (GPX), among others.

The protein αKlotho (hereafter referred as Klotho) has been found to be related to the appearance of oxidative stress. Thus, Klotho deficiency has been shown to increase endogenous ROS generation and accentuate oxidative stress [40,41]; conversely, Klotho administration effectively reduces oxidative stress and preserves mitochondrial function [42,43,44,45,46]. Klotho is an anti-aging transmembrane protein mainly expressed in kidney tubular cells where it plays an important role as a co-receptor for fibroblast growth factor 23 (FGF23). FGF23 suppresses renal inorganic phosphate reabsorption and active vitamin D biosynthesis [47]. A soluble form of Klotho can be also detected in blood, urine, and cerebrospinal fluid [48], and several studies relate the loss of soluble Klotho in plasma, and also in the kidney, with the progression of CKD [49], the pathogenesis of diabetic nephropathy [50], and cardiovascular disease (CVD) [51], and with metabolic and mineral-bone disorders [52,53]. The antioxidative effects of Klotho may involve the reduction of ROS through the expression of antioxidant proteins, and the suppression of ROS-related oxidative stress signaling pathways. In this sense, Klotho may promote the kidney transcription of Mn-SOD, catalase (CAT), heme oxygenase-1 (HO-1) and glutathione peroxidase (GPX) [43,54,55,56] through the activation of the forkhead box protein O transcription factor (FoxO) proteins [43,55,56] and the nuclear factor erythroid 2-related factor 2 (Nrf2) [57,58,59,60,61] (Figure 2).

## 2. The Klotho Protein

Klotho is a single-pass transmembrane protein mainly expressed in normal kidneys that was identified as an aging-suppressor protein more than two decades ago [48]. Klotho-null mice present shortened life span and display multiple phenotypes that resemble human premature aging: vascular calcification, infertility, emphysema, osteoporosis, skin atrophy, hair loss, thymic involution, osteopenia, motor neuron and hippocampal degeneration, and cognitive impairment [62]. This progeroid phenotype can be rescued by overexpressing Klotho via genetic manipulation or viral delivery [48,63]. Moreover, overexpression of Klotho extends the life span of mice, suppresses insulin signaling, and, interestingly, confers resistance against oxidative stress [48,63]. In humans, the levels of Klotho progressively decrease with age, being part of the progressive age-associated loss of kidney functions, and drastic reductions have been associated with many aging-related diseases, such as CKD, hypertension, cancer, diabetes, and cardiovascular disease [49,50,51,52,53,64]. In the kidneys, Klotho is highly present in the distal tubule, though it is also expressed in the proximal tubules, where it acts as a co-receptor for FGF23 [4], through which it plays a critical role in the regulation of phosphate and calcium homeostasis and in the synthesis of calcitriol (the active form of vitamin D).

Beyond the kidneys, Klotho is also expressed to a lesser extent in the brain, parathyroid glands, peripheral blood cells, and vascular tissue [65]; in addition to the membranous form, a soluble circulating form of Klotho is also present in blood, urine, and cerebrospinal fluid. Soluble Klotho arises from the proteolytic cleavage of the extracellular membrane portion by membrane-anchored proteases (a process called shedding), such as ADAM10 and ADAM17 [48,66]. Soluble Klotho acts as an endocrine or paracrine factor exerting anti-aging effects on multiple organs including the kidneys, bones, brain, heart, lungs, and endothelium. These effects include the suppression of growth factor signaling, regulation of several ion channels and transporters, and the suppression of oxidative stress [67]. The potential kidney-protective effects of Klotho include anti-inflammatory actions [68], the activation of autophagy [69], the preservation of the stemness of progenitor cells [46], the attenuation of oxidative stress [43,54,55,56,60,70], and the protection of mitochondrial function [45,56,71].

## 3. Antioxidative Effects of Klotho

Many pathways and mechanisms have been related to the antioxidative properties of Klotho. The first work pointing to this ability was published in 2005 by Yamamoto et al. [43]. The authors showed the ability of Klotho to confer resistance against oxidative stress in Klotho-overexpressing mice, which presented extended life (20–30% more), suppression of insulin signaling, increased Mn-SOD expression in muscles, less phosphorylated FoxOs, and lower levels of urinary 8-oxo-2′-deoxyguanosine, a marker of oxidative damage to DNA, than wild-type mice [43]. Klotho-overexpressing mice also survived significantly longer than wild-type mice after a sublethal dose of paraquat, an herbicide that generates superoxide [48], further indicating that Klotho overexpression enhances resistance to oxidative stress.

Subsequent studies have also suggested the potential relation of Klotho to oxidative stress. On one side, drugs with antioxidant potential such as statin, angiotensin II blockade, and N-acetylcysteine, increase Klotho expression [41,72,73,74,75]. On the other hand, oxidative stress decreases Klotho mRNA and protein in an inner medullary collecting duct (mIMCD3) mouse cell line [41]. In the same study, overexpression of the Klotho gene reduced the number of apoptotic cells after oxidant stress injury (exposure to hydrogen peroxide) [41]. In an immune-mediated glomerulonephritis mice model, increasing Klotho levels improved kidney function and reduced mitochondrial DNA fragmentation, superoxide anion generation, lipid peroxidation, and apoptosis, suggesting that Klotho may ameliorate mitochondrial oxidative stress [76].

The antioxidative effects of Klotho have been also determined in diabetic kidney disease, a serious complication of diabetes and the leading cause of end-stage kidney disease globally. Hyperglycemic conditions induce the generation of ROS that provokes dysfunction of kidney endothelial cells, further contributing to the progression of disease [77]. In glomerular endothelial cells, overexpression of Klotho significantly abolished, whereas Klotho knockdown enhanced, the injuries induced by glucose [50]. In *db*/*db* mice, a model of type 2 diabetes mellitus, intraperitoneal injections of recombinant Klotho reduced proximal tubular damage, the number of apoptotic tubular cells, and kidney ROS levels [71]. The reduction in ROS production was also observed in vitro in murine S1 proximal tubular cells incubated in high glucose conditions [71]. Along with the decreased ROS, Mitotracker Red analysis revealed increased mitochondria functionality in Klotho-treated S1 proximal tubular cells exposed to high glucose compared to untreated ones [71].

The antioxidant properties of Klotho are also evident in the uremic milieu, particularly in the endothelial cells. In patients with kidney disease, uremia raises oxidative stress and senescence in endothelial cells [78,79,80,81] and it has been demonstrated that extracellular Klotho prevents the senescence of endothelial cells induced by oxidative stress [55,78,82].

The upstream events mediating the induction antioxidative factors by Klotho and subsequent oxidative protection are not clear and may include to several factors and pathways. Hu et al. demonstrated that the protective effect of Klotho against hydrogen peroxide-induced cytotoxicity is partially abrogated by deletion of endogenous Erythropoietin receptor (EPOR), suggesting that EpoR is a downstream signaling component involved in the cytoprotective effect of Klotho [44]. The authors showed in vivo and in vitro that Klotho upregulates EpoR expression in the kidney, amplifying the EPO-triggered signaling pathways and protecting kidneys cells from oxidative injury [44]. Despite these findings, the investigation of the antioxidative effects of Klotho has been focused on the Klotho-mediated inhibition of insulin/insulin-like growth factor-1 (IGF-1) pathway, and on the activation of FoxO and Nrf2 proteins (Figure 2).

### 3.1. Klotho and FoxO Proteins

FoxO proteins consist of a subfamily of forkhead transcription factors with an important role in mediating the effects of insulin and growth factors on diverse physiological functions including the inhibition of cellular proliferation, the promotion of apoptosis, metabolism regulation, and protection against oxidative stress [83,84]. FoxO proteins are expressed in diverse organs and cell types including kidneys, liver, brain, muscles, pancreatic ß-cells, and adipocytes [85]. The FoxO transcription factors are negatively regulated by the insulin/IGF-1/PI3K/Akt signaling pathway. Akt-phosphorylated FoxO proteins remain in the cytoplasm and thus are unable to regulate gene expression. FoxO proteins in the nucleus stimulate the transcription of antioxidative enzymes, such as Mn-SOD and catalase, removing ROS [84]. Interestingly, soluble Klotho acts as an inhibitor of the intracellular insulin/IGF-1 signaling cascade. This activity likely contributes to the suppression of aging by Klotho, because inhibition of insulin-like signaling is an evolutionarily conserved mechanism for extending life span [86]. In fact, Klotho overexpression in mice extended the lifespan by repressing of insulin or IGF-1 signaling [48].

As mentioned above, transgenic overexpression of Klotho in mice and also the addition of soluble Klotho to cells led to increased Mn-SOD expression and decreased FoxO phosphorylation levels [43,44]. Recent studies suggest that this activation is mediated by the aforementioned ability of Klotho to inhibit the insulin/IGF-1/PI3K/Akt signaling cascade [43,48,56,87,88]. Thus, two consecutive studies determined that administration of recombinant Klotho decreased oxidative stress by promoting the expression of Mn-SOD via the insulin/IGF-1/PI3K/Akt/FoxO pathway in a nephrotoxicity model induced by the calcineurin inhibitor (CNI) FK-506 [56,87]. Oxidative stress caused by ROS is a common pathway in the CNI-induced kidney injury [89]. In the first of these works, Jin et al. [87] found that Klotho heterozygous mice were more susceptible to FK506-induced injury that was closely associated with aggravated oxidative stress and reduced Mn-SOD and nuclear FoxO expression. In a follow-up study, the same group focused on whether FK506-induced oxidative stress could be inhibited by exogenous treatment with the Klotho protein [56]. Klotho treatment dramatically downregulated the oxidative stress markers 8-hydroxy-2′deoxyguanosine (8-OHdG) and 4-hydroxy-hexenal (4-HHE). Moreover, the authors clearly demonstrated that Klotho inhibited PI3K/AKT-mediated phosphorylation of FoxO3a with the subsequent enhancement of FoxO3a binding to the Mn-SOD promoter. Indeed, Klotho increased Mn-SOD mRNA and protein expression in mitochondria during Fk506-induced toxicity in kidney cells in vitro. Thus, the authors suggested that Klotho increases resistance to oxidative stress in the kidneys via negative regulation of the PI3K/AKT pathway, and that FoxO3a-mediated Mn-SOD expression is involved in this process [56]. Conversely, the transfection of Klotho siRNA in cultured human embryonic kidney HEK293t cells abrogates this Klotho-mediated suppression of the insulin/IGF-1/PI3K/Akt pathway and thus FoxO activation [90].

The activation of ROS signaling and oxidative stress in aged brains is involved in the development of Alzheimer’s disease [91,92] and antioxidant-neuroprotective Akt-mediated effects of Klotho have been depicted [93]. Both exogenous and endogenous Klotho administration protected hippocampal neurons from glutamate and oligomeric amyloid ß-induced cytotoxicity, two oxidative stressors associated with increased ROS production. These effects were exerted through the inhibition of PI3K/AKT-mediated phosphorylation of FoxO proteins. Sustained inhibitory phosphorylation of FoxO was essential for the induction of the antioxidant enzymatic thioredoxin/peroxiredoxin (Trx/Prx) system [93]. In brains of aged SAMP8 mice, a model of aging which presents pathological features of Alzheimer’s disease that present increased phosphorylation of FoxO proteins [92], the administration of ligustilide, a proposed neuroprotective agent with antioxidant and antiapoptotic properties [90,93], attenuated oxidative stress and FoxO phosphorylation and increased the expression and activity of antioxidant enzymes compared with vehicle-treated control brains [94]. Interestingly, these effects were accompanied by increases in Klotho mRNA levels and protein expression in the choroid plexus and by increases in serum Klotho levels [94].

Moreover, it has been shown that treatment of HeLa, vascular endothelial, and Chinese hamster ovary (CHO) cultured cells with Klotho increases the resistance to oxidative stress through the downregulation of IGF-1/Akt signaling, and again, the reduction in phosphorylation of FoxO and the induction of Mn-SOD expression [43,55]. Thus, induction of Mn-SOD through the reduced phosphorylation of FoxO proteins seems to be important in the resistance to oxidative stress and the extended longevity of the Klotho-overexpressing mice [48], for the neuroprotective effects of Klotho administration in Alzheimer’s disease mice models [94], and for the antioxidative effect of Klotho in diverse cultured cells [43,94,95].

### 3.2. Nrf2

The appearance of oxidative stress in cells involves the activation of molecular pathways to remove ROS. The Keap1-Nrf2 system plays a central role in oxidative stress and is the most important endogenous antioxidant stress pathway found to date [22,96]. In response to oxidative stress and toxins, Nrf2 acts as an effective transcriptional activator of the antioxidant response element (ARE) located in the promoter region of Nrf2 target cyto-protective genes [97]. Kelch-like ECH-associated protein 1 (Keap1) is a negative regulator of Nrf2 under non-stress conditions; Keap1 binds to Nrf2, promoting its ubiquitination and proteasomal degradation. Under the influence of oxidative stress, the thiol Keap1 changes its conformation and dissociates from Nrf2. Released Nrf2 is then translocated to the nucleus to bind to ARE sequences, initiating the transcription of genes related to antioxidant defense, such as hemeoxygenase-1 (HO-1), NAD(P)H dehydrogenase (quinone 1) (NQO-1), and Mn-SOD among others [98,99]. Nrf2 impairment and ROS overproduction is common in aging tissues [98] and is a characteristic feature in CKD [14]. In several preclinical models, the activation of Nrf2 is clearly downregulated. In a model of murine subtotal nephrectomy, Nrf2 nuclear levels and the expression of Nrf2-target genes *Hmox-1, Cat,* and *Gpx4* were decreased in injured kidneys [100,101]. In unilateral ureteral obstruction models, Nrf2 is significantly downregulated in the chronic phase of the disease and the Nfr2-pathway inactivation is associated with sustained inflammation and damage progression [14]. Particularly, in diabetic nephropathy, there is a large body of evidence pointing to the protector role of Nrf2 against hyperglycemia-induced oxidative stress [102]. Decreased expression and/or activity of Nrf2 has been found in renal tissues of experimental animals or patients with diabetic nephropathy with a concomitant increase in ROS production. On the contrary, Nrf2 activation reduced oxidative damage and exerted protective effects against diabetic nephropathy [103,104].

The link between Klotho and Nrf2 had been established in studies using Klotho mutant mice [57]. Thus, Klotho-deficient mice exhibit decreased levels of both cytoplasmic and nuclear Nrf2 and, conversely, Klotho-overexpressing mice displayed increased nuclear levels of Nrf2 expression and activity [57]. Moreover, recent works in preclinical disease models point to the activation Nrf2 by Klotho as an important factor in the protection against kidney, cardiovascular, and neurological diseases [58,59,60,61]. Interestingly, Klotho deficiency upregulates the expression of Keap1 [105]; conversely, the inhibition of Keap1, with the subsequent activation of the Nrf2 pathway, significantly reduced kidney tissue aging in a Klotho deficiency mouse model [106].

Recombinant Klotho can activate the cellular protective Nrf2 pathway in cardiomyocytes [107], and in endothelial [108] human umbilical vein endothelial [109] and aortic smooth muscle cells [58]. Therefore, the activation of Nrf2 by Klotho seems to be an attractive therapeutic strategy against oxidative stress in kidney disease. Nevertheless, whether Klotho-conferred kidney protection is associated with Nrf2-mediated antioxidation remains unclear. In a recent study, Xing et al. provided the first evidence of Nrf2 signaling activation by Klotho in podocytes [60]. Moreover, the authors also demonstrated that this activation attenuated apoptosis of podocytes induced by high glucose levels/diabetes in vitro and in a diabetic *db*/*db* mouse model [60].

Klotho has been also suggested to protect the lung through the activation of endogenous antioxidative pathways [110]. Transfection of transmembrane or soluble Klotho or addition of Klotho protected cultured lung epithelial cells from oxidative stress by increasing the antioxidative capacity of the cells via the Nrf2 pathway [110]. Similarly, in an acute hyperoxic lung injury animal model, systemic elevation of Klotho alleviated oxidative damage and interstitial edema and stimulated an increase in total antioxidant capacity in the lung, possibly via Nrf pathways [110]. More recently, the downregulation of Klotho in mice has been related to an increase in ROS production, which activates the Nrf2/ARE signaling pathway and promotes the expression of fibrosis-related genes, thus accelerating renal fibrosis [111]. Conversely, overexpression of Klotho in a mice model of renal stone formation activated the Nrf2 signaling pathway, reducing oxidative damage and apoptosis in kidneys [112].

## 4. Klotho & Mitochondrial Dysfunction

As mentioned above, kidneys are highly metabolic organs with a high mitochondrial density and oxygen-consumption rates. The active reabsorption of sodium, glucose, and other ions from the filtered fluid results in a high demand for ATP. Therefore, the functionality of mitochondria is crucial for the maintenance of kidney health, and kidney disorders including AKI, kidney fibrosis, and diabetic nephropathy are highly related to mitochondrial dysfunction [113]. Klotho kidney health-protector and aging-suppressor functions could be related to protection from mitochondrial disfunction. Importantly, reductions in Klotho levels have been related with mitochondrial dysfunction [46]. Conversely, Klotho administration or expression enhancement improves mitochondrial dysfunction in CKD models [56], and Klotho-treated HK-2 cells exposed to FK506 and S1 proximal tubular cells exposed to high glucose revealed more functional mitochondria along with reduced ROS production when compared with non-treated cells [56,71]. Accumulated evidence suggests that this effect could be related to a Klotho-mediated modulation of mitochondrial uncoupling protein 1 (UCP1), B-cell lymphoma-2 (BCL-2), Wnt/β-catenin signaling pathway, and mitochondrial-related transcriptional regulators including peroxisome proliferator-activated receptor gamma coactivator 1-alpha (PGC-1 alpha), transcription factor EB, (TFEB) and peroxisome proliferator-activated receptor gamma (PPAR-gamma).

### 4.1. Mitochondrial Uncoupling Protein 1 (UCP1)

In muscle progenitor cells, age-related decline and genetic knockdown of Klotho drive progenitor cell mitochondrial dysfunction and impair muscle regeneration [46]. Mitochondrial dysfunction in these cells was derived from a pathologic ultrastructure, decreased mitochondrial bioenergetics, mitochondrial DNA damage, and increased senescence [46]. Klotho knockout mice also present diminished expression of mitochondrial uncoupling protein 1 (UCP1) [114]. UCPs are a family of mitochondrial anion carrier proteins expressed in the mitochondrial inner membrane that can act as proton channels, leading to proton re-entry into the mitochondrial matrix from the intermembrane space, thus collapsing the proton gradient and decreasing the membrane potential. This decrease causes a reduction in ROS emission from the electron transport chain, uncoupling respiration from ATP production [115]. Although UCP1 has been depicted as a critical factor for thermogenesis and is predominantly found in brown fat mitochondria, where it is responsible for the generation of heat rather than ATP [116], several studies have demonstrated that UCP1 may also have an important role as a modulator of ROS generation in mitochondria of brown adipose tissue, thymus, and importantly, in kidneys [117]. In normal kidneys, UCP1 is located in the tubular epithelial cells, where it reduces oxidative stress in ischemia and cisplatin-induced AKI models [117].

### 4.2. B-Cell Lymphoma-2 (BCL-2)

As discussed above, Klotho administration ameliorated mitochondrial dysfunction in an FK506-induced nephropathy mice model and in FK506-treated HK-2 cells [56]. Klotho administration increased basal respiration function, ATP-linked respiration, maximal respiration, and spare respiratory capacity, and reduced mitochondrial ROS formation through the inhibition of the PI3K/AKT pathway [56]. In addition, the authors found a decrease in FK506 treatment-related mitochondria-associated apoptosis by Klotho treatment via B-cell lymphoma-2 (Bcl-2) upregulation [56]. Anti-apoptotic Bcl-2 proteins reside on the outer mitochondrial membrane and prevent apoptosis by inhibiting the activation of the pro-apoptotic family members Bax and Bak. These proteins can commit a cell to its programmed death by permeabilizing the outer mitochondrial membrane and subsequent initiation of the caspase cascade. In addition, antiapoptotic Bcl-2 proteins inhibit Beclin 1-dependent autophagy. Some authors have suggested a kidney-protective effect of Klotho by disrupting the beclin 1/Bcl-2 complex, upregulating autophagy activity and protecting against IRI-induced acute kidney injury in mice [118,119].

### 4.3. Wnt/β-Catenin Signaling

Wnt/β-catenin signaling activation plays a crucial role in mediating mitochondrial dysfunction and age-related renal fibrosis [120]. Klotho is an endogenous antagonist of Wnt/β-catenin signaling pathway [121] whose activation has been observed in a variety of CKD experimental models, predominantly in damaged renal tubular epithelial cells [121,122,123,124]. The activation of Wnt/β-catenin signaling triggers tubular epithelial cell transition to mesenchymal or senescent phenotype, and promotes renal fibrosis and is associated with mitochondrial dysfunction [16,120]. Conversely, the inhibition of Wnt/β-catenin significantly protected normal structures and functions of mitochondria, and mitigated age-related renal fibrosis, suggesting the role of Wnt/β-catenin signaling in mediating age-related renal fibrosis and its association with mitochondrial dysfunction [120].

Miao et al. [45] assessed the protective effects of Klotho in CKD mice and in kidney tubular epithelial cells. In an ischemia-reperfusion injury CKD mouse model, ectopic overexpression of Klotho by gene delivery preserved kidney function and inhibited kidney fibrosis [45]. Moreover, Klotho significantly preserved mitochondrial mass, inhibited mitochondrial ROS production, and restored the expression of mitochondrial respiration chain complex subunits in the kidneys [45]. The same authors found similar effects of treatment with recombinant human Klotho on mitochondrial function in cultured kidney proximal tubular epithelial cells. Importantly, Klotho significantly inhibited Wnt1 and Wnt9a-induced mitochondrial injury in kidney tubular cells, suggesting that the protective effects of Klotho on mitochondria may be mediated by the inhibition of Wnt/β-catenin signaling [45].

### 4.4. Peroxisome Proliferator-Activated Receptor Gamma Coactivator 1-Alpha (PGC1alpha)

Recombinant soluble Klotho protein administration in *db*/*db* mice confers protection from diabetic kidney injury through reducing ROS and directly protecting mitochondrial functionality [71]. This protection was evidenced by a reduction in the number of damaged mitochondria in the renal cortex when compared to untreated mice [71]. The mitochondrial protection was related with the kidney activation of the adenosine monophosphate-activated protein kinase (AMPK)/peroxisome proliferator-activated receptor gamma coactivator 1-alpha (PGC1alpha) pathway [71]. AMPK, known as the guardian of mitochondrial homeostasis [125], activates PGC1alpha, which is a master regulator of mitochondrial biogenesis [126]. Importantly, many studies have shown that AMPK protects against renal dysfunction [127], and that its level declines with aging [128]. A plausible upstream activator of AMPK in response to Klotho is liver kinase beta 1 (LKB1) [129]. Klotho also increased OXPHOS and NAD+. The NAD+ is a pivotal cofactor involved in many metabolic pathways, including the AMPK-PGC1alpha pathway. NAD+ has recently emerged as a powerful kidney-protective molecule [130].

### 4.5. Transcription Factor EB (TFEB)

A recent work has suggested that Klotho promotes both the expression of the transcription factor EB (TFEB) and TFEB-mediated lysosomal gene transcription [131]. TFEB is a master regulator of lysosomal biogenesis, and therefore of the autophagy–lysosomal pathway, that removes misfolded protein aggregates or damaged organelles. Under baseline conditions, phosphorylated TFEB is retained in the cytosol. Under adverse conditions, TFEB is dephosphorylated and migrates to the nucleus where it promotes the expression of lysosomal biogenesis genes by binding to the coordinated lysosomal expression and regulation (CLEAR) element [132]. FK506 significantly reduces the protein levels of TFEB in nephropathy mice model and in HK-2 cells [129]. Recombinant Klotho administration to FK506-induced injury HK-2 cells increases TFEB-mediated gene transcriptional activity in the CLEAR element [132]. The upregulation of TFEB by Klotho could explain some of the described beneficial effects of this protein in the FK506-induced nephropathy models [56,87,131]. The activated TFEB pathway has been described to promote lysosomal degradation of damaged mitochondria [133,134]. However, TFEB can also promote mitochondrial biogenesis by promoting PGC-1 expression and the expression of antioxidant genes such as HO-1, SOD2, and thioredoxin 1 [133]. These results suggest that Klotho-induced TFEB regulation may be useful for the management of FK506-induced nephrotoxicity by modulating oxidative stress and protecting mitochondrial functionality.

### 4.6. Peroxisome Proliferator-Activated Receptor Gamma (PPAR-γ)

PPAR-γ is a member of the nuclear receptor superfamily of ligand-inducible transcription factors, abundantly expressed in adipocytes, and to a lesser extent, in the kidneys [135]. Therapeutic approaches based on the modulation of PPAR-γ activity have attenuated or even prevented CKD [136]. Animal models of insulin resistance, type 2 diabetes, and hypertension, as well as clinical studies in type 2 diabetic patients, have demonstrated kidney protection after treatment with thiazolidinediones, a synthetic PPAR-γ agonist antidiabetic drug, observing an improvement in albumin excretion, and in glucose and lipid profile, and in high glucose-treated HK-2 cells, reduced proximal tubular cell proliferation and the expression of TGF-β and MCP-1 [136,137]. In the post hoc analysis of Prospective Pioglitazone Clinical Trial in Macrovascular Events (PROactive) study, which included 5154 patients with DM2 and macrovascular disease, pioglitazone treatment reduced major cardiovascular endpoints in patients with CKD (*n* = 597) compared to placebo, but in patients without CKD, the effect of pioglitazone versus placebo was similar regardless of eGFR; taking all patients into account, pioglitazone treatment reduced the annual decline in eGFR compared to placebo [138]

In addition to its known participation in the regulation of energetic metabolism, anti-inflammatory and antifibrotic properties related to the increased expression of Klotho are attributed to it, contributing and promoting the Klotho protective functions, suggesting a link between PPAR-γ and Klotho [139,140].

In mouse kidneys, HEK293 cells, and several renal epithelial cell lines, PPAR-γ overexpression mediated by treatment with thiazolidinedione, adenovirus, cilostazol, or pioglitazone (PPAR-γ agonists) increased Klotho expression, while PPAR-γ antagonist treatment attenuated it [139,141].

The contribution of PPAR-γ to the delay in natural and CKD-associated aging would be related to the regulation of mitochondrial function by blocking the RAAS, and the results suggest the participation of Klotho in this process. In a study with 5/6 nephrectomy rats, the antifibrotic Klotho and PPAR-γ were reduced in the remaining kidney, and losartan, an angiotensin II (AngII) receptor antagonist, restored their levels. The Ang II administration to Madin–Darby canine kidney (MDCK) cell culture induced a decrease in both Klotho and PPAR-γ, and this effect was mitigated by losartan, suggesting that the effect of Ang II downregulating Klotho is mediated by PPAR-γ [140].

## 5. Klotho Replacement as a Possible Antioxidant and Mitochondria-Protective Strategy

Any therapy based on the administration, restoration, or stimulation of exogenous Klotho might provide a novel strategy for combating aging and age-associated diseases including kidney disease. To date, no human studies of Klotho protein administration have been performed. In contrast, preclinical data clearly support the therapeutic potential of soluble Klotho protein for age-related disorders and Klotho deficiency-associated diseases. Diverse animal studies point to the administration of exogenous Klotho as a safe and effective approximation to reduce oxidative stress [142]. In Klotho-deficient mice, Klotho gene delivery effectively rescues many phenotypes observed, prolonging life span [143]. Similarly, Klotho gene delivery attenuates the progression of hypertension and kidney damage in spontaneous hypertensive rats [144,145], and ameliorates angiotensin II-induced kidney injury [146], improves endothelial function [147], and protects from uremic cardiomyopathy in heterozygous Klotho-deficient CKD mice [148].

The administration of soluble Klotho protein also confers protection against multiple disorders. Thus, soluble Klotho protein attenuates kidney damage and preserves kidney function in an ischemia–reperfusion injury model causing acute kidney injury, which is a state of acute Klotho deficiency [142]. Furthermore, Klotho protein inhibited renal fibrosis in a unilateral ureteral obstruction kidney-injury model [149]. Interestingly, intraperitoneal injection of soluble Klotho protein effectively extended the life span of homozygous Klotho-deficient mice, ameliorated premature aging-related phenotypes, such as growth retardation, premature thymus involution, and vascular calcification, and effectively reduced cellular senescence [150].

The stimulation of endogenous Klotho production can also present beneficial effects in some circumstances. This approximation could be of high clinical relevance, given the absence of the implementation of gene delivery and Klotho protein-based therapies for clinical use. Specifically, Klotho could be an interesting antioxidative and mitochondria-protective treatment. Interestingly, diverse drugs with antioxidative potential already marketed increase Klotho expression in vivo and in vitro [72,73,74,75]. Some of these drugs are PPARγ agonists [139,151,152], the angiotensin II type I receptor antagonists (losartan) [75,153,154,155], HMG-CoA reductase inhibitors (statin) [152], vitamin D active derivatives [155,156,157,158], and pentoxifylline [159].

## 6. Conclusions

Klotho is known as an aging suppressor, and mitochondrial dysfunction and oxidative stress are the hallmarks of aging. Oxidative stress is a main contributor to both acute and chronic conditions including kidney disease. In recent years, the role of Klotho in kidney disease has attracted increasing attention [160,161,162,163]. Some of the results exposed in this review point to the existence of Klotho’s protective effects against oxidative stress, promoting the expression of antioxidant proteins, such as Mn-SOD, catalase, HO-1, and GPX, among others, through the inhibition of FoxO phosphorylation induced by the IGF-1/PI3K/Akt signaling pathway, and the activation of the Nrf2-ARE system mediated by Keap1 inhibition. In addition, Klotho, through the modulation of proteins such as the aforementioned UCP1, BCL-2, Wnt/β-catenin, PGC-1 alpha, TFEB, and PPAR-γ, promotes the proper mitochondrial function of vital importance in organs that support high metabolic activity, as is the case of the kidneys, and has protective activity against renal fibrosis induced by activation of the Wnt/β-catenin pathway.

However, the upstream events that mediate the induction of antioxidant factors by Klotho and the subsequent oxidative protection are not clear and may include several factors and pathways, so further studies are needed in this regard. Despite the enlightening results regarding the protective role of Klotho as an anti-aging, antioxidant, mitochondrial function-protecting, oxidative stress-reducing, antifibrotic, anti-inflammatory, autophagy-stimulating, and stemness of progenitor cells-preserving molecule, obtained in experimental models of premature aging associated with various pathologies, and in particular kidney disease, it is necessary to design clinical trials in order to transfer the results obtained from experimental models to clinical practice and to be able to analyze its potential efficacy in kidney protection.

The discovery of new therapeutic approaches that slow down the progression of lesions and improve the quality of life of kidney patients continues to be a challenge for nephrologists and the scientific community. Large-scale studies have demonstrated kidney-protective effects for glucagon-like peptide-1 receptor agonists (GLP-1 RA) and SGLT2 inhibitors (SGLT2i), attracting great attention in recent years. The underlying mechanisms by which GLP-1 RA and SGLT2i provide this protection are not fully understood. It has been proposed that the beneficial effects of these drugs may be partly mediated by antioxidative actions [164,165]. Thus, GLP1 RA have shown protection against oxidative stress, cellular senescence, and chronic inflammation in aging-related diseases [165]. Similarly, the administration of SGLT2i ameliorated oxidative stress in experimental models of diabetes, diabetic nephropathy, and septic AKI [164,166]. Interestingly, both GLP-1 RA and SGLT2i promote the expression and/or prevent the downregulation of Klotho in different tissues and cell types [167,168]. SGLT2i treatment increased Klotho availability in type 2 diabetic patients with poorly controlled diabetes and early diabetic kidney disease, as well as in stressed tubular cells [168].

Taking into account the effects of these drugs on the production of Klotho and the close relationship between Klotho and oxidative stress, especially in CKD, we believe that future clinical–experimental studies are worthwhile in order to analyze the potential role of Klotho in the antioxidative properties of GLP-1 RA and SGLT2i.

## Data Availability

Not applicable.
